# Navigating the Lead Paradox: Successful Co‐Implantation of Cardiac Contractility Modulation Device and a Micra Leadless Pacemaker

**DOI:** 10.1111/jce.70244

**Published:** 2026-01-09

**Authors:** Gabriele Pavani, Paolo Garrone, Gianpaolo Varalda, Antonino Previti, Matteo Bianco, Alessandra Chinaglia

**Affiliations:** ^1^ Cardiac Electrophysiology Unit San Luigi Gonzaga University Hospital Orbassano, Turin Italy; ^2^ Interventional Cardiology Unit San Luigi Gonzaga University Hospital Orbassano, Turin Italy; ^3^ Cardiology Department San Luigi Gonzaga University Hospital Orbassano, Turin Italy; ^4^ University of Turin Turin Italy

**Keywords:** cardiac amyloidosis, Cardiac Contractility Modulation, case report, device–device interaction, electrophysiology technique, leadless pacemaker

## Abstract

**Background:**

Wild‐type transthyretin cardiac amyloidosis (ATTRwt‐CA) can lead to refractory heart failure. A “lead paradox” occurs when patients with a Micra leadless pacemaker require lead‐based Cardiac Contractility Modulation (CCM) therapy.

**Case Summary:**

We detail the first co‐implantation of CCM and Micra devices in a 78‐year‐old male with ATTRwt‐CA and NYHA III heart failure. A multi‐view fluoroscopic technique ensured spatial separation, while specific device programming mitigated electrical crosstalk post‐procedure.

**Conclusion:**

At 1 year, the patient stabilized to NYHA Class II without further hospitalizations. This dual‐device strategy is a feasible and safe technical roadmap for this complex clinical problem.

## Graphical abstract

This central illustration visually summarizes the first co‐implantation of a Cardiac Contractility Modulation (CCM) system and a Micra leadless pacemaker in a 78‐year‐old male with wild‐type transthyretin cardiac amyloidosis and NYHA Class III heart failure. The patient's case presented a “lead paradox”—a need for a lead‐based therapy (CCM) in an individual with a pre‐existing leadless solution (Micra). The procedural strategy emphasized a multi‐view fluoroscopic technique to ensure spatial separation and post‐implant device programming to mitigate interaction. At 1‐year follow‐up, the patient's condition improved to NYHA Class II with no further heart failure hospitalizations. The case establishes a feasible and safe technical roadmap for this dual‐device strategy.

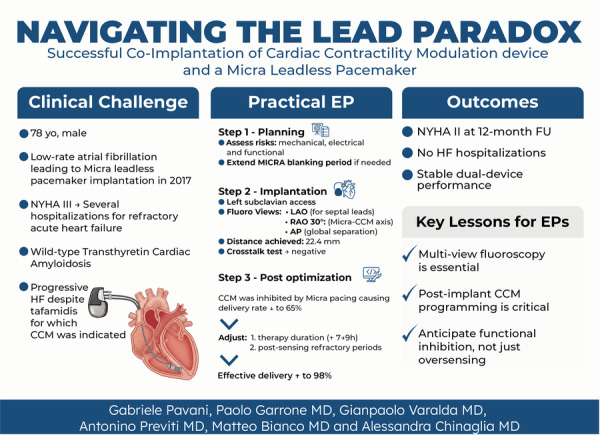

## 1 Introduction: The Clinical Challenge

Wild‐type transthyretin cardiac amyloidosis (ATTRwt‐CA) is increasingly recognized as a cause of heart failure, particularly in older adults. The disease involves the relentless infiltration of the myocardium by misfolded proteins, resulting in ventricular thickening, restrictive physiology, and, ultimately, intractable heart failure [1]. While disease‐modifying therapies like tafamidis can slow progression, many patients with advanced ATTRwt‐CA remain highly symptomatic, presenting a significant management challenge [2]. Additionally, at the time of this patient's treatment, reimbursement for tafamidis was not available for patients with NYHA Class III heart failure in our country (Italy), further limiting therapeutic options. Furthermore, arrhythmias and conduction system disease are frequently encountered in patients with cardiac amyloidosis [3].

For these patients, two distinct device therapies have emerged to address different aspects of their condition. Cardiac Contractility Modulation (CCM) offers a novel approach for symptomatic heart failure, delivering non‐excitatory electrical signals to improve myocardial function through gene expression changes. The FIX‐HF‐5C trial validated its use in patients with reduced ejection fraction (HFrEF) and a narrow QRS complex [4]. At the same time, the Micra leadless pacemaker has become an essential tool for managing bradyarrhythmias, as its design avoids the lead‐ and pocket‐related complications inherent to transvenous systems—a benefit demonstrated with a low rate of major complications reported at 6 months [5] and further supported by lower reintervention rates at 2 years in high‐risk subgroups [6].

A clinical dilemma arises when a patient needs both: this creates a “lead paradox” where a lead‐based therapy (CCM) is indicated in a patient who specifically required a leadless pacing solution. The technical feasibility and safety of combining these two intracardiac devices have not been previously described. With the ongoing AMY‐CCM registry (NCT05167799) set to clarify the role of CCM in amyloidosis, the challenge of managing patients with pre‐existing leadless pacemakers will likely become more common [7].

Initial pilot data from this registry reinforce CCM's potential in this population. A study of 10 patients with advanced ATTR‐CM found that CCM therapy significantly reduced heart failure hospitalizations by 86% and improved functional status from NYHA Class III/IV to II. Critically, this improvement allowed all patients to become eligible for tafamidis, establishing CCM as a potential bridge to disease‐modifying therapy and justifying its use in complex cases [8].

## 2 Case Presentation

Our patient was a 78‐year‐old male with a history of ATTRwt‐CA, diagnosed in 2023, and permanent atrial fibrillation. He had a Micra leadless pacemaker implanted in 2017 at another institution for symptomatic low‐rate atrial fibrillation. This leadless approach was reportedly chosen due to the patient's very thin body habitus to mitigate the risk of device pocket erosion. Despite being on tafamidis and optimized medical therapy, his heart failure progressed, with an LVEF of 30%–35% and persistent NYHA Class III symptoms, leading to recurrent hospitalizations. The percentage of ventricular pacing was less than 20%, so we could exclude a pacemaker‐induced cardiomyopathy. Given his refractory condition, he was enrolled in the AMY‐CCM clinical trial.

## 3 The Practical Technique: A Step‐By‐Step Approach

### 3.1 Step 1: Pre‐Procedural Planning and Risk Mitigation

The primary challenge was the potential for negative device‐device interaction. Our pre‐procedural planning focused on three key risks:

1. **Mechanical interference:** The physical interaction between the new CCM leads and the indwelling Micra device during and after implantation.
2. **Electrical crosstalk:** The primary concern was that the high‐voltage signal artifact from the CCM device could be erroneously sensed by the Micra outside of its programmed refractory period, leading to inappropriate pacing inhibition—a life‐threatening risk in a pacemaker‐dependent patient.
3. **Functional conflict:** A secondary concern was that the CCM device might be inhibited by the Micra's pacing output, reducing therapy delivery.

To mitigate these risks, we consulted with the device manufacturer, who confirmed a small number of successful but unpublished co‐implantations. We noted that the Micra's programmable post‐ventricular blanking periods could be extended, providing a safeguard against oversensing. Our definitive strategy was to achieve maximal, stable spatial separation between the devices.

### 3.2 Step 2: The Implantation Procedure (Figure 1)

**Figure 1:**
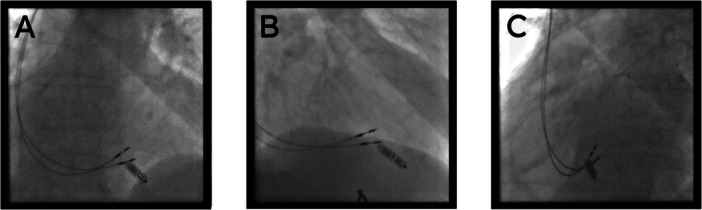
Fluoroscopic Images of Co‐implanted Cardiac Contractility Modulation (CCM) and Micra devices. Final fluoroscopic images demonstrating the relative positions of the two CCM active‐fixation leads and the previously implanted Micra leadless pacemaker. (A) Anteroposterior (AP) view showing the spatial separation between the septally‐placed CCM leads and the apically‐positioned Micra. The maximum distance between the devices was measured at 22.4 mm. (B) Right anterior oblique (RAO) 30° view. (C) Left anterior oblique (LAO) view, used to confirm the septal placement of the CCM leads.

The procedure was performed under conscious sedation with antibiotic prophylaxis.

- **Access and pocket creation:** A standard left pectoral incision was made, and venous access was gained via the left subclavian vein. As the patient had a leadless pacemaker, the standard contralateral approach, which is usual for dual‐device implants, was not necessary.
- **Lead positioning:** This was the most critical phase. Two 58 cm active‐fixation leads (Tendril STS, Abbott), which function as the right ventricular (RV) lead and the local sense (LS) lead for the CCM system, were advanced into the right ventricle. We used a multi‐view fluoroscopic approach:
  ◦ LAO view (steep): This projection was essential to confirm that the leads were truly on the interventricular septum, separating them from the free wall.
  ◦ RAO 30° view: This view allowed for clear visualization of the leads' basal‐apical axis in relation to the apically sited Micra.
  ◦ AP view: This provided the final confirmation of the overall lead position and separation.

The goal was to place the two CCM leads on the mid‐interventricular septum, at a safe distance from the Micra. After several manipulations, a stable position was achieved with a measured maximum distance of 22.4 mm between the distal CCM lead and the Micra tines.

- **Parameter check and fixation:** With the leads in their final position, electrical parameters were verified. Sensing was good (4.0 mV on both leads), and impedances were stable (RV lead 368 Ω, LS lead 542 Ω). The leads were then securely sutured to the fascia.
- **Final connection and testing:** The leads were connected to the CCM generator (Optimizer Smart Mini, Impulse Dynamics). Before closing the pocket, a crucial crosstalk test was performed. The CCM was programmed to maximum output (7.5 V) while we simultaneously monitored the Micra's real‐time electrogram and marker channels via its programmer. No oversensing or pacing inhibition was observed. The generator was then placed in the pocket, and the incision was closed. The total procedure time was 90 min.

### 3.3 Step 3: Follow‐Up and Optimization

Initial follow‐up revealed an important practical lesson. Device interrogation showed that frequent ventricular pacing from the Micra was inhibiting the CCM system, resulting in suboptimal therapy delivery (only 65% of the programmed time).

This functional interaction was addressed with a two‐part programming adjustment:

1. The total daily **duration of CCM therapy was increased** from 7 to 9 h. This simple change successfully raised the *effective* therapy delivery to 98% within the new, longer window.
2. The CCM system's sensing parameters, specifically the **post‐sensing refractory periods**, **were increased** to be less susceptible to inhibition from the paced ventricular events.

At the 1‐year follow‐up (May 2025), the patient's condition had improved and stabilized in NYHA Class II. He has had no further hospitalizations for heart failure, and his diuretic dose has been successfully reduced.

Objectively, he showed significant improvement in multiple parameters from his baseline (April 2024) prior to CCM implantation: LVEF improved from 30% to 35% (peaking at 39% at 3 months), and his 6‐min walk test distance more than doubled from 150 to 365 m. Diastolic function also improved markedly, with the average E/E′ ratio decreasing from 27 to 15. Pulmonary artery systolic pressure was reduced from 50 to 40 mmHg. His quality‐of‐life score (KCCQ) remained stable (70 at baseline, 70 at 1‐year).

All device parameters remain stable. The Micra pacing percentage increased to over 88% (attributed to the natural progression of his underlying conduction system disease) without any inappropriate inhibition, and the CCM delivered consistent therapy.

## 4 Discussion: Lessons for the Electrophysiologist

This case provides a practical roadmap for the co‐implantation of a CCM device and a Micra leadless pacemaker. Several key learning points emerge for the practicing electrophysiologist:

- **Feasibility:** This dual‐device therapy is technically feasible and, with careful planning, appears safe.
- **Procedural technique is key:** Success relies on a meticulous implantation technique. The use of multi‐view fluoroscopy (especially steep LAO and RAO) is not just helpful but essential for ensuring adequate spatial separation, which is the primary defense against electrical crosstalk.
- **Anticipate post‐implant interactions:** While oversensing by the Micra was our main pre‐procedural concern, the more significant issue was the inhibition of the CCM by the Micra. Clinicians should anticipate this functional interaction and be prepared to optimize CCM programming post‐implant by adjusting therapy duration and sensing parameters. Further adjustments could be necessary at follow‐up, due to an increase in ventricular pacing percentage.
- **High pacing burden and CRT:** An important consideration at follow‐up was the high percentage of ventricular pacing (> 88%), which was due to progression of the patient's conduction disease. About 20% of patients with a high burden of ventricular pacing are known to develop pacemaker‐induced cardiomyopathy, but this patient did not progress to this condition. On the contrary, his LVEF improved (30%–35%) despite an increase in RV pacing, his NYHA Class improved (III–II), and his 6MWT distance doubled. Therefore, an upgrade to CRT was not indicated. Furthermore, adding a CRT device with a coronary sinus lead and a standard RV lead to a complex system already containing two CCM leads and a Micra would have significantly increased procedural risk and the risk of infection and hardware complications.
- **A growing clinical need:** The AMY‐CCM registry will soon provide large‐scale data on the clinical effectiveness of CCM in amyloidosis. As the therapy is adopted for this population, electrophysiologists will increasingly encounter patients with pre‐existing leadless pacemakers. Our experience provides a proven technical framework to manage this specific scenario.

## 5 Conclusion

Co‐implantation of a CCM device and a Micra leadless pacemaker is a viable and safe option for patients with advanced heart failure and complex arrhythmic needs. This case report, the first of its kind, provides a procedural roadmap, emphasizing the importance of pre‐procedural planning and precise, fluoroscopically‐guided lead placement. As the findings from the AMY‐CCM registry clarify the therapeutic role of CCM in cardiac amyloidosis, this report will serve as a valuable technical adjunct, providing a proven strategy for the subset of patients with co‐existing leadless pacing needs.

## Conflicts of Interest

The authors declare no conflicts of interest.
